# Messengers and Messages for Tweets That Used #thinspo and #fitspo Hashtags in 2016

**DOI:** 10.5888/pcd15.170309

**Published:** 2018-01-04

**Authors:** Jenine K. Harris, Alexis Duncan, Vera Men, Nora Shevick, Melissa J. Krauss, Patricia A. Cavazos-Rehg

**Affiliations:** 1 Brown School, Washington University in St. Louis, St. Louis, Missouri; 2 Department of Psychiatry, School of Medicine, Washington University in St. Louis, St. Louis, Missouri; 3 Washington University in St. Louis, St. Louis, Missouri

## Abstract

**Introduction:**

Twitter is widely used by young adults and is popular for seeking and sharing health information. The hashtags #thinspo and #fitspo provide a way to identify tweets designed to inspire thinness (thinspiration, thinspo) or fitness (fitspiration, fitspo). However, despite having different purposes, both terms may be associated with content that promotes eating disorders. We sought to 1) examine and compare the characteristics of senders and the content of tweets using these hashtags and 2) identify characteristics associated with engagement with a #thinspo or #fitspo tweet.

**Methods:**

In May 2016 we collected 1,035 tweets with #thinspo and #fitspo hashtags by using a constructed week sampling procedure. Using consensus coding, pairs of raters assessed each tweet’s topic and associated images and videos. We used descriptive statistics to examine topics and user characteristics and inferential models to determine topics and characteristics associated with retweets, likes, and replies to tweets.

**Results:**

Of the 1,035 tweets, 696 (67.2%) were relevant to body image, fitness, food, dieting, or eating disorders. Fitspo tweets came from organizations or businesses, were promotional, and focused on nutrition and exercise, whereas #thinspo tweets came from individuals, focused on thinness and disordered eating behaviors, and contained images of extremely thin women. Rates of retweeting and liking were significantly higher for #thinspo than for #fitspo.

**Conclusion:**

Characteristics of messages and messengers differed between #thinspo and #fitspo tweets; #thinspo tweets were used for messages about disordered eating. Public health professionals should consider using the #thinspo hashtag to reach the #thinspo group.

## Introduction

Disordered eating behaviors are common, particularly among adolescent and young adult women, with rates in these groups as high as 16% for binge eating, 20% for purging compensatory behaviors (vomiting, laxatives, diuretics, diet pills), and 61% for nonpurging compensatory behaviors (fasting, skipping meals, restricting) (1,2). These behaviors are associated with leading causes of morbidity and mortality, including depression, alcohol misuse, and obesity (1,3). The age of onset for eating disorders is typically the late teens or early twenties (4).

As of 2015, 89% of US teenagers aged 13 to 17 and 86% of US adults aged 18 to 29 used social media; 33% of 13- to 17-year-olds and 36% of online 18- to 29-year-olds used Twitter (5,6). The percentages of teenaged and young adult Twitter users who are males and nonwhite are higher than percentages of these groups in the general population, and young adults who use Twitter are more likely to be college graduates than young adults in the general population (6,7). Twitter is an application for “microblogging,” or sending and receiving brief (140 characters) messages or “tweets.” Twitter accounts can be followed by other Twitter users, allowing individuals or organizations to receive and share or “retweet” messages to followers. Twitter users can also “like” a tweet by clicking on a heart, enter a reply that will show beneath the original tweet if clicked on, or mention another Twitter user by including @username in a tweet; the tweet with the mention will then appear in the mentioned account. Hashtags are used on Twitter for the formation of groups interested in particular topics or events (8) and make it easier to find tweets and like or share them (9).

Facebook is more widely used than Twitter, and older adults and parents are less likely to monitor the use of Twitter by adolescents and young adults than they are to monitor use of Facebook (10,11), affording young users greater anonymity. This anonymity may increase sharing of information on Twitter on such health issues as disordered eating behaviors or struggles with body image (12). Evidence of changes in health behaviors resulting from exposure to and use of social media is mixed (13,14); however, approximately 80% of US adult internet users have searched online for health information (15,16) and 70% report being influenced by what they found (17).

The quality of health information on social media varies widely; one study found that about half of “healthy living” blogs contained negative and stigmatizing messages about food or weight (18). Because of high rates of social media use among adolescents and young adults, concern is increasing about the promotion of disordered eating behaviors among online communities (19,20). Viewing images of extremely thin people may alter an individual’s views on healthy body image and weight and contribute to normalization of disordered eating behaviors (21). Social media users with disordered eating behaviors who feel isolated may be especially vulnerable to these messages as they seek a sense of belonging (22).

Thinspiration and thinspo are terms used on social media to identify messages as inspiration for weight loss (23,24). Likewise, fitspiration and fitspo are used when posting messages to inspire people to exercise and eat healthy food (24–26). Although intended to focus on healthy behavior, fitspo has been described as similar to thinspo in promoting unhealthy behaviors (24,25). To better understand social media users who send messages and engage with #thinspo and #fitspo, we 1) examined and compared senders and content of tweets using the #thinspo and #fitspo hashtags, and 2) identified characteristics associated with engagement with #thinspo and #fitspo tweets.

## Methods

In May 2016 we used a constructed week sampling procedure (27,28) to collect tweets that included #thinspo or #fitspo. The constructed week includes one Monday, one Tuesday, and so forth during a specified time to account for variation in volume and topics on different days and during different times of a month. We initially collected data on 2 constructed weeks (14 days) of tweets (n = 28,941) for an average of 2,067 #thinspo or #fitspo tweets daily. From the 2 weeks we selected a single week and took a random sample of 100 #thinspo and 50 #fitspo tweets per day for coding. We used #thinspo and #fitspo instead of #thinspiration and #fitspiration after an informal review of hashtags found the shorter versions were used more often. We sampled fewer tweets from #fitspo after we found #thinspo tweets were more commonly sent. For each tweet, we collected data on the number of followers of the sender, the number of users followed by the sender, and the number of tweets the sender had ever sent. To determine engagement with the #thinspo and #fitspo tweets, approximately one year after the tweets in our data were sent, we collected the number of retweets, likes, and replies to each tweet.

Initial data were collected by using NodeXL (Social Media Research Foundation) (29), a plug-in for Excel (Microsoft Corp) that accesses Twitter and downloads tweets based on key words, hashtags, Twitter user names, and other information. To collect the number of retweets, likes, and replies, we opened each tweet manually and recorded the information. The sampling process resulted in 700 tweets collected with the #thinspo hashtag and 354 tweets collected with the #fitspo hashtag. A small number (n = 18) of duplicate tweets with both hashtags and one tweet with neither hashtag were removed. The final sample size for coding was 1,035 unique tweets: 692 #thinspo tweets and 343 #fitspo tweets from 562 unique Twitter user accounts.

### Coding tweets

The codebook was developed by using an iterative process. We started the codebook by including codes consistent with related studies examining thinspiration or fitspiration social media content (24–26). The initial codebook included items related to the focus of the tweet text and the focus of images, videos, and websites linked from the tweet. We also included codes for whether the tweet appeared promotional (ie, promoting a product, organization, or business), whether it was in English, whether the text of the tweet was only hashtags, and whether the Twitter account the tweet came from appeared to belong to a person or an organization or business. After 3 authors (A.D., N.S., V.M.) coded a sample of 50 tweets, we met to discuss modifications to the codebook. The modified codebook was then used by the 3 authors for 2 rounds of reliability coding on approximately 5% of the data; after each round, the authors held a group discussion to clarify codes with low reliability. The Fleiss κ for the final round of reliability coding was almost perfect (0.81–1.0) for 17 of the 43 codes, fair to substantial agreement (0.41–0.80) for 11 codes, and below 0.41 for 15 codes. Because we did not reach at least moderate agreement for all codes, for the final coding we used consensus coding, whereby 2 coders classified each tweet and met to discuss and arrive at consensus. Each of 3 coders was assigned approximately two-thirds of the tweets; each tweet was coded by 2 coders.

After reliability testing and discussion, the following 10 final text topic codes were included: eating less, eating healthy, exercise, gaining strength, losing weight, binging, purging (vomiting or using laxatives or diuretics), wanting a body type or body part characteristic (eg, thigh gap), mentions of a type of eating disorder, and mentions of a medication. Each tweet could be coded for more than one topic. Image and video codes were as follows: images or video focuses on food, body or parts of a body, or something else; number of images or videos; number of images or videos that show a male, a female, or “can’t tell [sex of person shown]”; number of images or videos that show a person who is extremely skinny or skeletal, muscular, or neither; number of images or videos that show butt, thighs–legs, arms, neck–shoulders, torso (shoulders to hips), waist–stomach–hips, or full body; number of images that show healthy food like fruits, vegetables, grilled meat, or unhealthy food like candy, chips, fried food, or a combination of healthy and unhealthy food. Web URLs in tweets were clicked and the main topic of the web page was coded as dieting, healthy eating, physical activity, medication–vitamins–supplements, something else. Mentions were coded by searching each tweet for the @ symbol and coding tweets with @ as including a mention.

### Data analysis

The 562 unique Twitter users in the 1,035 tweet data set included 403 users (71.7%) who contributed one tweet and 159 users who contributed 2 to 23 tweets. Because of multiple tweets from 159 Twitter users, the data failed the assumption of independent observations, so we used descriptive statistics to examine topics and characteristics for all tweets and to compare #thinspo and #fitspo tweets.

Few tweets included an image in any one category (eg, torso, arms), resulting in medians for the number of images per category being 0 or near 0. We recoded the number of images variables to binary variables for whether or not each tweet included each image category and reported frequencies and percentages for tweets containing each type of image.

To determine which topics and characteristics were associated with retweets, likes, and replies, we examined the distribution of these variables and selected an appropriate statistical model. All 3 variables failed a Shapiro–Wilks test of normality. Just 94 tweets had any replies, and the distributions of the retweet and likes variables were extremely right skewed with variances far above their means. We recoded the replies variable as a binary variable: no replies and one or more replies.

Because past research demonstrated that the characteristics of Twitter users influence retweeting, liking, and replying to tweets (9), we used 2-level models with tweet characteristics at level 1 and Twitter user characteristics at level 2. We used 2-level negative binomial regression to predict retweeting and likes and 2-level logistic regression to predict replies. Models included Twitter user-level and tweet-level characteristics associated with retweeting, liking, and replying to tweets (ie, engaging with tweets) in prior studies, including number of tweets and followers (9) and the type of Twitter user (30). Although hashtag and URL inclusion in a tweet influences engagement (9), all tweets included at least one hashtag because of the method of data collection, and none of the #thinspo tweets included URLs, so we did not include hashtag or URL variables in the models. There is no evidence we are aware of that including an image or video in a tweet increases engagement, so we did not include this variable in the models. There is mixed evidence on whether including a mention decreases engagement (31) or has no influence (9) on engagement, so we included mentions in the model. Finally, we added a predictor representing whether the tweet included #thinspo or #fitspo. We used Akaike information criterion (AIC) to compare model fit and interpreted better-fitting models (ie, models with lower AIC). Clean data and R commands for analyses are available at https://github.com/coding2share/Research-project-code/tree/master/thinspo-fitspo. Alpha was set at .05. The study was approved by the institutional review board at Washington University in St. Louis (IRB 201611145).

## Results

Of the 1,035 tweets, 696 were relevant to body image, fitness, food, dieting, or eating disorders; 15 tweets were not relevant and 324 were inaccessible (n = 315) or had content that was unclear (n = 9). Most relevant tweets were in English (Table 1). Thinspo tweets had higher medians than #fitspo tweets for numbers of retweets and likes. Only 94 (13.5%) tweets received replies: 17.9% of #thinspo tweets and 5.0% of #fitspo tweets. Of 458 #thinspo tweets, 141 (30.8%) were hashtags only, while 21 of 238 (8.8%) #fitspo tweets were hashtags only. Most (98.1%) hashtags-only tweets included images or videos.

**Table 1:** Characteristics of Relevant #thinspo and #fitspo Tweets Collected From Twitter During a Constructed Week^a^, May 2016

Characteristic	No. (%)^b^		
	#thinspo Tweets (n = 458)	#fitspo Tweets (n = 238)	All Tweets (n = 696)
**Engagement with tweet**			
Retweets, median (IQR)	20 (9-45)	0 (0–0)	8 (0–31)
Likes, median (IQR)	25 (9.25–53)	0 (0–1.0)	9.5 (1–34)
Replies	82 (17.9)	12 (5.0)	94 (13.5)
**Tweet features and topics**			
Tweet is in English	397 (86.7)	232 (97.5)	629 (90.4)
Tweet text is hashtags only	141 (30.8)	21 (8.8)	162 (23.3)
Tweet is an advertisement or a fitness/diet tip that appears promotional	1 (0.2)	164 (68.9)	165 (23.7)
**Tweet includes a link to a website**	0	140 (58.8)	140 (20.1)
Linked website is about dieting	0	115 (48.3)	115 (16.5)
Linked website is about healthy eating	0	3 (1.3)	3 (0.4)
Linked website is about physical activity	0	8 (3.4)	8 (1.1)
Linked website is about something else	0	18 (7.6)	18 (2.6)
Link is broken	0	8 (3.4)	8 (1.1)
**The tweet includes a mention**	223 (48.7)	67 (28.2)	290 (41.7)
**The tweet includes links to images/videos**	447 (97.6)	178 (74.8)	625 (89.8)
**Images/videos focus on food**	0	32 (13.4)	32 (4.6)
Images show healthy food	0	19 (8.0)	19 (2.7)
Images show unhealthy food	0	2 (0.8)	2 (0.3)
Images show a combination of healthy/unhealthy food	0	11 (4.6)	11 (1.6)
**Images/videos are a body or parts of a body**	435 (95.0)	114 (47.9)	549 (78.9)
Full body	243 (53.1)	44 (18.5)	287 (41.2)
Waist–stomach–hips	155 (33.8)	8 (3.4)	163 (23.4)
Torso	161 (35.2)	28 (11.8)	189 (27.2)
Neck–shoulders	71 (15.5)	27 (11.3)	98 (14.1)
Arms	36 (7.9)	6 (2.5)	42 (6.0)
Thighs–legs	161 (35.2)	2 (0.8)	163 (23.4)
Butt	12 (2.6)	0	12 (1.7)
**Image/video includes one or more person who appears to be . . .**			
Extremely skinny or skeletal	346 (75.5)	8 (3.4)	354 (50.9)
Muscular	3 (0.7)	45 (18.9)	48 (6.9)
**Image/video includes one or more person who appears to be . . . **			
Male only	0	15 (6.3)	15 (2.2)
Female only	435 (95.0)	91 (38.2)	526 (75.6)
Both	0	7 (2.9)	7 (1.0)
Unable to tell	0	1 (0.4)	1 (0.1)
**The Twitter user discourages or makes fun of doing something unhealthy or extreme**			
Yes	2 (0.4)	1 (0.4)	3 (0.4)
No	399 (87.1)	230 (96.6)	629 (90.4)
Neutral or unable to tell	57 (12.4)	7 (2.9)	64 (9.2)

Abbreviation: IQR, interquartile range.

a Constructed week defined as one Sunday, one Monday, one Tuesday, one Wednesday, one Thursday, one Friday, and one Saturday chosen at random from a specified period of time.

b Unless otherwise indicated.

More than half of #fitspo tweets were promotional and/or included a link to a website, while just one #thinspo tweet was promotional, and none included a link to a website. A small number (n = 32) of #fitspo tweets focused on food images, while no #thinspo tweets focused on food images. Most (95.0%) #thinspo tweets included images or videos showing a body or body part; only 47.9% of #fitspo tweets did so. About one-third (33.8%) of #thinspo tweets included one or more images or videos focused on the waist–stomach–hips, while only 3.4% of #fitspo tweet images/videos did so. Most (75.5%) #thinspo tweets included an image showing an extremely skinny or skeletal person; only 3.4% of #fitspo tweets included such an image. In addition, 18.9% of #fitspo tweets included images/videos of a muscular person, while muscular people were featured in 0.7% of #thinspo tweets. Of the 45 #fitspo tweets with muscular images, 26 were of females only, 13 images were of males only (out of the 15 #fitspo tweets with males-only images), 5 were of both males and females, and one image was of a body that was not clearly male or female. Nearly all (95%) #thinspo tweet images were of females (Table 1).

The percentages of tweets on each topic differed for #thinspo and #fitspo (Figure). Compared to #thinspo tweets, a higher percentage of #fitspo tweets contained the topics of exercise, strength, eating less, eating healthy, and medication. #Thinspo tweets had higher percentages than #fitspo tweets of the topics of losing weight, wanting a certain body type or body part characteristic, eating disorders, binging, and purging.

**Figure:**
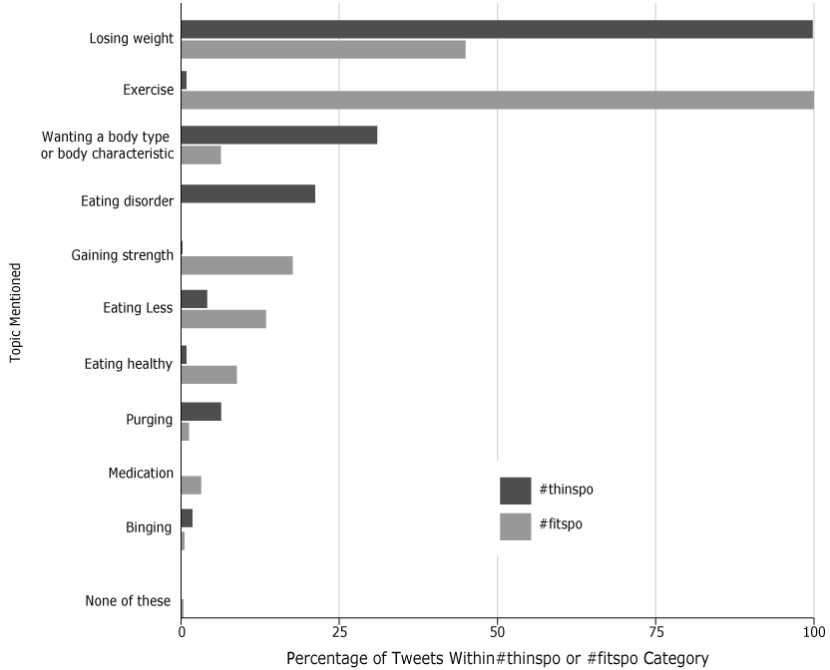
Percentage of topics mentioned in a sample of #thinspo or #fitspo tweets, May 2016. Percentages add to more than 100 because each tweet could be coded for multiple topics. **Table:** 

Topic	#fitspo, %	#thinspo, %
None of these	0	0.2
Binging	0.4	2.0
Medication	2.9	0
Purging	1.3	6.3
Eating healthy	8.8	0.9
Eating less	13.4	4.1
Gaining strength	17.6	0.2
Eating disorder	0	21.2
Wanting a body type or body part characteristic	6.3	31.0
Exercise	100.0	0.9
Losing weight	45.0	99.8

The #fitspo tweeters had nearly twice the number of followers that #thinspo tweeters had (Table 2). Likewise, #fitspo tweeters tweeted more overall than did #thinspo tweeters, while #thinspo tweeters favorited more tweets (Table 2). Some of these differences may be explained by the higher percentage of #fitspo tweeters (54.4%) than #thinspo tweeters (0.3%) who appeared to represent organizations or businesses; a greater percentage of #thinspo tweeters (99.7%) than #fitspo tweeters (35.0%) appeared to be individuals.

**Table 2:** Characteristics of 390 Twitter Users Who Tweeted 696 Relevant #thinspo and #fitspo Tweets Collected During a Constructed Week^a^, May 2016

Characteristic	#thinspo Tweets (n = 287)	#fitspo Tweets (n = 103)	All Tweets (n = 390)
**Category, median (IQR)**			
Twitter users followed	212.5 (92.25–544.25)	212 (77.5–829)	212 (88–592)
Followers of Twitter users	267 (104.25–718.25)	489 (132.5–1,299.5)	308 (110–870)
Tweets	1,591.5 (425.25–5,841.75)	3,378 (675–17,061.5)	1,794 (507–7,105)
Favorites	585.5 (92.0–2,319.75)	44 (3.5–370.5)	363 (48–1,745)
**Type of Twitter user, n (%)**			
Individual representing personal views	286 (99.7)	36 (35.0)	322 (82.6)
Organization or business	1 (0.3)	56 (54.4)	57 (14.6)
Unable to tell	0	11 (10.7)	11 (2.8)

Abbreviation: IQR, interquartile range.

a Constructed week defined as one Sunday, one Monday, one Tuesday, one Wednesday, one Thursday, one Friday, and one Saturday chosen at random from a specified period of time.

The retweet model with the #thinspo–#fitspo variable (AIC = 3,694.6) was a better fit than the model without (AIC = 3,813.6). In the better-fitting model, retweets were positively and significantly associated with tweeting #thinspo compared with #fitspo (Table 3). The Twitter user appearing to be an individual representing personal views was also positively and significantly associated with retweets, while the number of tweets from the account was negatively and significantly associated with retweets. The number of followers and the “unable to tell” user type were not associated with retweets.

**Table 3:** Multilevel Models With the Best Fit for Predicting the Number of Retweets, Likes, and the Probability of a Reply to a Tweet With the #thinspo or #fitspo Hashtag^a^ on Twitter

Characteristic	No. of Retweets		No. of Likes		Probability of Reply	
	b (SE)	*P* Value	b (SE)	*P* Value	b (SE)	*P* Value
**Constant**	−1.22 (0.31)	<.001	0.27 (0.25)	.28	−10.80 (2.58)	<.001
**Tweet characteristic **						
Contains mention	0.47 (0.13)	<.001	0.36 (0.13)	.004	−0.06 (0.72)	.93
Contains #thinspo	3.48 (0.31)	<.001	2.38 (0.27)	<.001	—	—
**Sender characteristic**						
No. of followers, in 100s	0.007 (0.005)	.15	0.007 (0.005)	.16	0.004 (0.02)	.88
No. tweets sent, in 100s	−0.0007 (0.0002)	<.001	−0.0007 (0.0002)	<.001	−0.0004 (0.001)	.77
**Type of Twitter user**						
Organization or business	1 [Reference]		1 [Reference]		1 [Reference]	
Individual representing personal views	0.85 (0.40)	.03	0.67 (0.33)	.04	1.58 (2.51)	.53
Unable to tell	0.52 (0.48)	.27	0.03 (0.40)	.94	−0.33 (4.37)	.94

Abbreviation: SE, standard error.

a Models are based on 574 tweets and 335 tweet senders.

The likes model with the #thinspo–#fitspo variable was a better fit (AIC = 4,055.2) than the model without the #thinspo–#fitspo variable (AIC = 4,125.8). Consistent with the retweet model, the “unable to tell” user type and the number of followers were not significantly associated with likes; however, using the #thinspo hashtag and appearing to be an individual representing personal views were both positively and significantly associated with the number of likes (Table 3). The number of tweets sent by the Twitter user was negatively and significantly associated with likes. Finally, the model without the #thinspo–#fitspo variable was a slightly better fit (AIC = 359.6) than the same model with the #thinspo–#fitspo variable (AIC = 361.5) at explaining replies. However, none of the predictors were significant in either model (Table 3).

## Discussion

We examined 1,035 tweets that used the #thinspo and #fitspo hashtags from a constructed week in May 2016. Although a recent study found similarities in content on thinspiration and fitspiration websites (24), we found differences in messages, messengers, and engagement between tweets containing #thinspo and tweets containing #fitspo.

The topics of #thinspo tweets were different from the topics of #fitspo tweets: #fitspo tweets were more likely to include the topics of exercise, strength, healthy eating, eating less, and medication and #thinspo tweets were more likely to include the topics of binging, purging, losing weight, eating disorders, and wanting a certain body type or body part characteristic. 

In addition to having different messages, #thinspo and #fitspo tweets had differences in messengers. Consistent with the promotional content, #fitspo tweets were more likely than #thinspo tweets to be sent by organizations or businesses or people representing them. With only one exception, #thinspo tweets were sent by accounts that appeared to belong to an individual representing personal views. Also consistent with #fitspo tweeters being organizations or businesses, those who sent #fitspo tweets had more followers and sent more tweets than those who sent #thinspo tweets.

We also found differences in engagement: #thinspo tweets had significantly more retweets and likes than #fitspo tweets, which is contrary to expectations and prior research (9), given the larger number of followers and tweets sent by #fitspo tweeters. Even after the #thinspo–#fitspo variable was added to the model, the number of followers of a Twitter user was negatively associated with likes, suggesting that people using these hashtags are liking tweets from individuals rather than from organizations or businesses. Taken together with the characteristics of the messenger, it appears that Twitter users using the #thinspo hashtag may be a community of individuals coalescing around the thin body image while #fitspo tweeters are businesses promoting or selling fitness-related products.

Although the link between social media consumption and health behavior is not yet clear, engaging with social media content through retweeting or liking a tweet may have some influence on behavior (13,14). The #thinspo tweets were positively and significantly associated with retweets and likes, and the content focused on unhealthy behaviors and images. Meanwhile, #thinspo tweeters mostly represented personal views. These characteristics suggest an engaged community sharing information that normalizes disordered eating behaviors (21) and increases body dissatisfaction and disordered eating behaviors (23). Although eating disorder–focused advocacy organizations, research centers, and treatment facilities are active on Twitter, they do not often use hashtags in their messages. Our findings suggest that these organizations may reach more people in need of intervention for disordered eating behaviors if they use the #thinspo hashtag. Organizations may also wish to consider a strategy in which employees tweet from separate accounts to take advantage of higher levels of engagement with individual accounts compared with organizational accounts.

Our study has several limitations. First, our data collection strategy included #thinspo and #fitspo, which may have excluded tweets with the longer #thinspiration and #fitspiration hashtags. In addition, although the constructed week strategy of collecting tweets increased the likelihood that our data were representative, the selected tweets may not be representative of the population of #thinspo and #fitspo tweets. Despite these limitations, this study contributes to our understanding of how #thinspo and #fitspo are used on social media by identifying differences in the messengers, messages, and engagement that could aid public health practitioners and researchers in identifying and contacting people at risk for disordered eating behavior. Future studies might examine whether changes in knowledge, attitudes, or behaviors would result from sending replies or direct messages about healthy eating or positive body image to Twitter users who tweet the #thinspo hashtag.
